# Comparison of lumefantrine, mefloquine, and piperaquine concentrations between capillary plasma and venous plasma samples in pregnant women with uncomplicated falciparum and vivax malaria

**DOI:** 10.1128/aac.00093-24

**Published:** 2024-04-10

**Authors:** Makoto Saito, Pornpimon Wilaisrisak, Mupawjay Pimanpanarak, Jacher Viladpai-Nguen, Moo Kho Paw, Urairat Koesukwiwat, Joel Tarning, Nicholas J. White, Francois Nosten, Rose McGready

**Affiliations:** 1 Shoklo Malaria Research Unit, Mahidol-Oxford Tropical Medicine Research Unit, Faculty of Tropical Medicine, Mahidol University, Mae Sot, Thailand; 2 Centre for Tropical Medicine and Global Health, Nuffield Department of Medicine, University of Oxford, Oxford, United Kingdom; 3 Division of Infectious Diseases, Advanced Clinical Research Center, Institute of Medical Science, University of Tokyo, Tokyo, Japan; 4 Mahidol Oxford Tropical Medicine Research Unit (MORU), Faculty of Tropical Medicine, Mahidol University, Bangkok, Thailand; The Children's Hospital of Philadelphia, Philadelphia, Pennsylvania, USA

**Keywords:** malaria, pregnancy, capillary, venous, lumefantrine, mefloquine, piperaquine, desbutyl-lumefantrine, carboxy-mefloquine

## Abstract

Capillary samples offer practical benefits compared with venous samples for the measurement of drug concentrations, but the relationship between the two measures varies between different drugs. We measured the concentrations of lumefantrine, mefloquine, piperaquine in 270 pairs of venous plasma and concurrent capillary plasma samples collected from 270 pregnant women with uncomplicated falciparum or vivax malaria. The median and range of venous plasma concentrations included in this study were 447.5 ng/mL (8.81–3,370) for lumefantrine (day 7, *n* = 76, median total dose received 96.0 mg/kg), 17.9 ng/mL (1.72–181) for desbutyl-lumefantrine, 1,885 ng/mL (762–4,830) for mefloquine (days 3–21, *n* = 90, median total dose 24.9 mg/kg), 641 ng/mL (79.9–1,950) for carboxy-mefloquine, and 51.8 ng/mL (3.57–851) for piperaquine (days 3–21, *n* = 89, median total dose 52.2 mg/kg). Although venous and capillary plasma concentrations showed a high correlation (Pearson’s correlation coefficient: 0.90–0.99) for all antimalarials and their primary metabolites, they were not directly interchangeable. Using the concurrent capillary plasma concentrations and other variables, the proportions of venous plasma samples predicted within a ±10% precision range was 34% (26/76) for lumefantrine, 36% (32/89) for desbutyl-lumefantrine, 74% (67/90) for mefloquine, 82% (74/90) for carboxy-mefloquine, and 24% (21/89) for piperaquine. Venous plasma concentrations of mefloquine, but not lumefantrine and piperaquine, could be predicted by capillary plasma samples with an acceptable level of agreement. Capillary plasma samples can be utilized for pharmacokinetic and clinical studies, but caution surrounding cut-off values is required at the individual level.

**CLINICAL TRIALS:**

This study is registered with ClinicalTrials.gov as NCT01054248.

## INTRODUCTION

Capillary blood samples are considered as a potential alternative to venous samples for assessing drug concentrations. In resource-limited settings, capillary samples are preferred because they are less expensive, easier to obtain, and less invasive than venous samples, especially in small children. In malaria clinical trials, capillary samples have the additional advantage that capillary samples are collected routinely for quantitating malaria parasitemia. In pregnancy, capillary blood is also routinely used for monitoring maternal anemia during antenatal care in the tropics. Therefore, in addition to the practical and operational advantages in study settings (e.g., pharmacokinetic studies), the potential advantage of capillary blood sampling over venous sampling is that it can be used for clinical management in the field (1). Previously, the venous antimalarial drug concentration on day 7 has been used as a strong predictor of treatment failure (i.e., recrudescence) (2). This can also be assessed using capillary blood samples. There have been a few studies directly comparing venous and capillary blood antimalarial drug concentrations (3–9) and only one cohort included pregnant women (3, 7). We assessed how precisely and reliably venous plasma drug concentrations of three major antimalarials, namely lumefantrine, mefloquine, and piperaquine, could be predicted by using capillary plasma drug concentrations in pregnant women.

## MATERIALS AND METHODS

### Study design and eligibility

This study was a part of a population pharmacokinetic study, which was nested in an open-label, randomized, controlled trial of three artemisinin-based combination therapies (ACTs) in pregnancy conducted in 2010–2016 on the Thailand-Myanmar border (NCT01054248). Details of study design and clinical outcomes were reported elsewhere (10, 11). Pregnant women with uncomplicated malaria (either falciparum, vivax, or mixed infection of both) were eligible. There was no restriction on gestational age at enrollment and ultrasound was used for confirming pregnancy and viability of fetus. Malaria infection was confirmed by microscopy.

### Intervention

Enrolled women were randomly allocated to one of the three ACTs using sealed envelopes: dihydroartemisinin-piperaquine (DP), artesunate-mefloquine (ASMQ), or extended regimen artemether-lumefantrine (AL+). DP was given at the standard dose (2.4 mg/kg dihydroartemisinin with 20 mg/kg piperaquine once daily for 3 days), rounded to the nearest half tablet (40 mg/320 mg dihydroartemisinin/piperaquine per tablet manufactured by Holley Pharmacy, China). ASMQ was given at the standard dose (once daily for 3 days) either as loose doses of artesunate (4 mg/kg/day) and mefloquine (8.3 mg/kg/day) or fixed dose (artesunate 200 mg with mefloquine hydrochloride 440 mg each day, manufactured by Far-Manguinhos, Brazil). The loose dose was rounded to the nearest quarter of a tablet for artesunate (50 mg/tablet, Guilin, China) and mefloquine (250 mg/tablet, Atlantic Laboratories Corp., Thailand). AL+ was given as an extended regimen: five tablets (20/120 mg artemether/lumefantrine per tablet, Novartis, Switzerland) twice per day for 4 days (at 0, 8, 24, 36, 48, 60, 72 and 84 h), with 250 mL of chocolate milk containing 7 g of fat for each dose. All doses were fully supervised.

### Blood sampling

Capillary blood (200 µL) was taken from a finger prick in four hematocrit tubes on day 3 (day 4 for AL+) and day 7 for all eligible women, and additional samples were taken randomly at different days between day 3 and day 21. Venous blood was taken concurrently. Capillary samples were collected in sodium heparin hematocrit tubes centrifuged at 11,000 × *g* for 3 minutes. Venous samples were collected in lithium heparin tubes and centrifuged at 2,000 × *g* for 10 minutes. Blood samples were centrifuged immediately at each site, and plasma was kept in cryotubes at −80°C at the central laboratory in Mae Sot, Thailand.

### Drug concentration measurement

Drug concentration in plasma was measured at the Pharmacology Laboratory of the Mahidol Oxford Tropical Medicine Research Unit, using the high-performance liquid chromatography tandem mass spectrometry, as described previously (12, 13). The overall method performance for each drug is summarized in Table S1. Samples were excluded from the analysis if the measured venous drug concentration was below the quantification level: the lower limit of quantification was 7.77 ng/mL for lumefantrine, 0.808 ng/mL for desbutyl-lumefantrine, 7.64 ng/mL for carboxy-mefloquine, 7.64 ng/mL for mefloquine, and 1.20 ng/mL for piperaquine.

### Statistical method

In this analysis comparing antimalarial concentrations between capillary plasma and venous plasma, 90 pairs of venous and capillary samples from 90 women were randomly selected for each drug using *random* command in Stata MP 16.1 (StataCorp, TX, USA). Analyzed samples were randomly selected from days 3–21 samples for DP and ASMQ, whereas for AL+, all analyzed samples were taken on day 7 (144–196 hours) (2) because day 7 lumefantrine concentration is widely used for predicting the treatment outcome (1, 2, 4, 14, 15).

Firstly, the agreement between the venous and capillary samples were visualized by Bland and Altman plots (16, 17). Logarithmic transformation was used if there was a relationship between the difference between two sample type concentrations and the concentration itself (17). Pearson’s correlation coefficient was used for describing the linear correlation of venous and capillary drug concentrations, and paired *t*-test was used assessing the null hypothesis that venous and capillary plasma samples were equal. Apparent outliers on the plot were excluded from statistical tests and regression models. Secondly, multivariable linear regression models including other variables were built for predicting the drug concentrations in venous plasma samples. Venous plasma concentration (with or without natural logarithmic transformation) was used as the dependent variable. Explanatory variables included capillary plasma drug concentration, sampling time from the first dose, estimated gestational age, hematocrit, initial parasitemia load, presence of parasitemia, maternal age, height, and weight. Data on the sampling date were used for estimated gestational age, hematocrit, and presence of parasitemia. Fractional polynomials were used to describe the non-linear relationship with continuous variables (e.g., sampling time). Adjusted coefficient of determination (*R*^2^) was used for selecting the best predictive model (3). The agreement between observed and predicted venous drug concentrations was illustrated by residual plots and quantitatively assessed by mean absolute difference relative to the observed value, which was calculated as


$$\;\;\frac{1}{n}\sum \frac{|Predicted\;value-Observed\;value|}{Observed\;value}\times 100\left(\%\right).$$

## RESULTS

### Study inclusion and baseline characteristics

**Fig 1:**
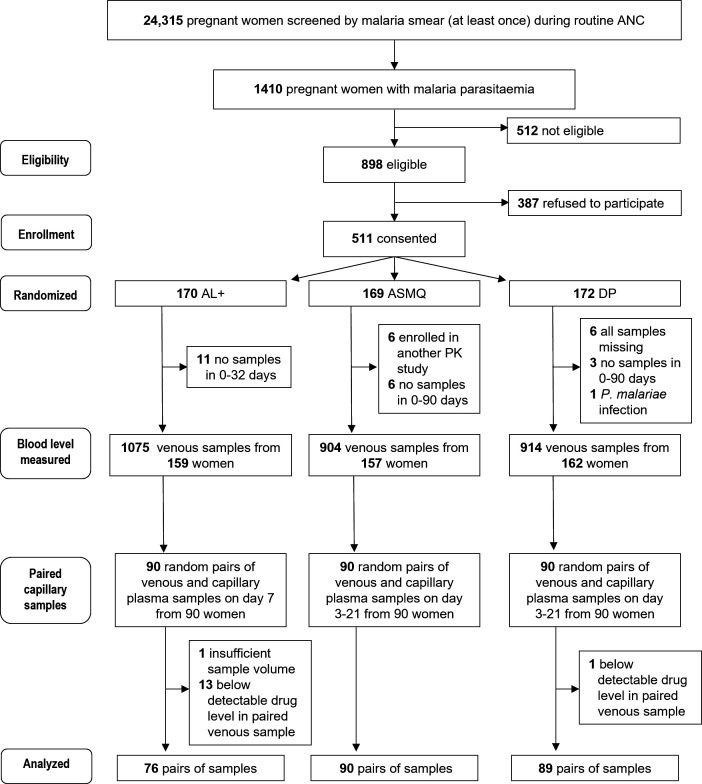
Participant flow in the randomized comparison of extended regimen artemether-lumefantrine (AL+), artesunate-mefloquine (ASMQ), and dihydroartemisinin-piperaquine (DP).

In total, 511 women were enrolled and randomized into one of the three ACTs. Among the 477 women with at least one eligible venous plasma sample, this study randomly selected 90 capillary plasma samples from 90 women in each treatment arm (Fig. 1). After measuring drug concentrations, 15 samples were excluded (14 in AL+ arm and one in DP arm) because there was insufficient sample volume (*n* = 1 in AL+) or undetectable venous plasma concentrations (*n* = 13 in AL+ and *n* = 1 in DP).

The baseline characteristics were similar among the three treatment arms (Table 1): the median age ranged from 23.5 to 24.0 years, and the median estimated gestational age at drug measurement ranged from 26.0 to 28.0 weeks. Since all AL+ samples were selected from day 7 samples, no women were parasitemic on the day the samples were taken. In contrast, 4% (4/89) of DP and 7% (5/90) of ASMQ samples were parasitemic (all on day 3).

**TABLE 1:** Demographic and clinical information of pregnant women whose venous and capillary plasma samples were measured for antimalarial drug concentrations^*a*^^,^^*c*^

	LUM (*n* = 76)	MFQ (*n* = 90)	PIP (*n* = 89)
Age (years)	23.5 (18–40)	24 (18–45)	23 (18–41)
EGA^*b*^ (weeks)	26.4 (9.8–40.1)	28.0 (11.5–39.7)	26.0 (9.4–40.6)
Parity	1 (0–9)	1 (0–13)	1 (0–8)
Malaria species on day 0			
Pf mono-infection	28% (21)	20% (18)	22% (20)
Pv mono-infection	68% (52)	77% (69)	76% (68)
Pf + Pv	4% (3)	3% (3)	1% (1)
Presence of malaria^*b*^			
No parasitemia	100% (75)	93% (71)	96% (85)
Pf	0% (0)	7% (5)	3% (3)
Pv	0% (0)	0% (0)	1% (1)
Hematocrit^*b*^ (%)	30 (20–42) (*n* = 75)	31 (24–47) (*n* = 68)	31 (21–40) (*n* = 71)
Weight (kg)	50 (39–65)	52 (31–67)	50 (36–77)
Height (cm)	149.5 (140-162)	151 (137–165)	150 (138–164)
Sampling time from the first dose (hours)	164.9 (157.8–185.2)	162.1 (48.0–501.2)	162.9 (48.0–502.3)
Dose of total partner drug/body weight (mg/kg)	96.0 (72.7–123.1)	24.9 (19.7–34.7)	52.2 (48.0–58.5)

^*a*^ Median (range) or percentage (number) is shown.

^*b*^ On the day when the drug concentration was measured.

^*c*^ EGA, estimated gestational age; LUM, lumefantrine; MFQ, mefloquine; Pf, *Plasmodium falciparum*; PIP, piperaquine; Pv, *Plasmodium vivax*.

The median and range of venous plasma drug concentrations included in this study were 447.5 ng/mL (8.81–3,370) for lumefantrine, 17.9 ng/mL (1.72–181) for desbutyl-lumefantrine, 1,885 ng/mL (762–4,830) for mefloquine, 641 ng/mL (79.9–1,950) for carboxy-mefloquine, and 51.8 ng/mL (3.57–851) for piperaquine. The median sampling time from the first dose was similar among three treatment arms (162.1–164.9 hours).

### Direct comparison of venous and capillary plasma antimalarial drug concentrations

**Fig 2:**
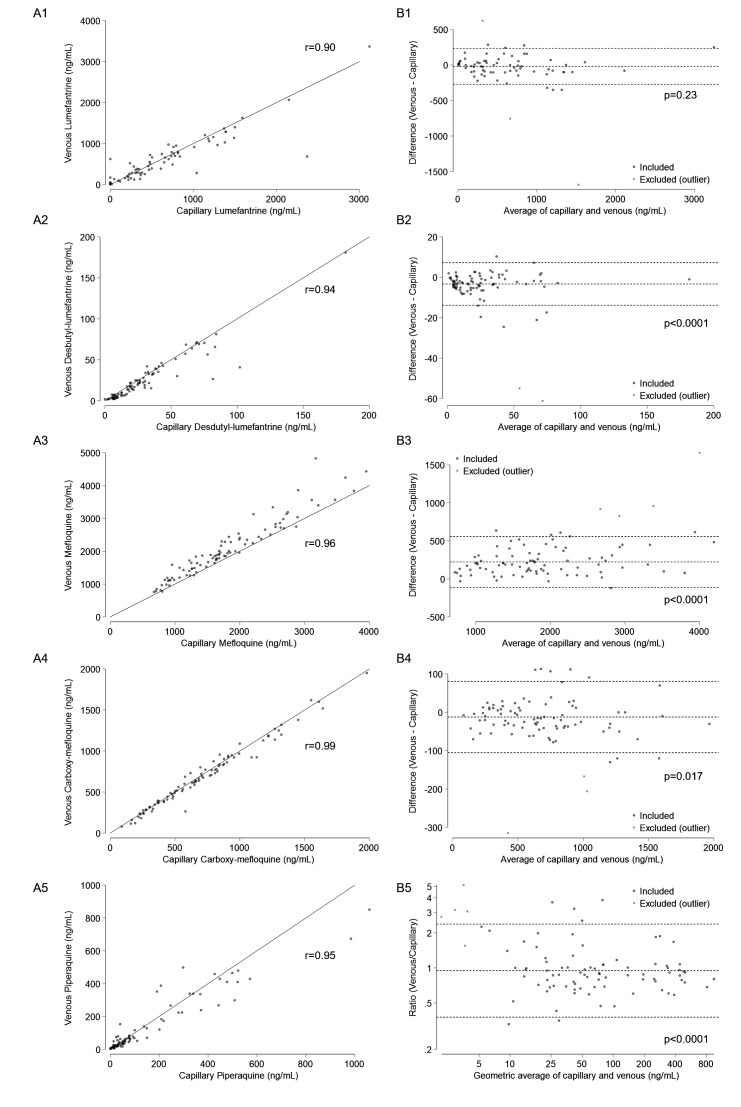
Relationship between venous and capillary plasma concentrations for each antimalarial drug. (**A**) Scatterplots of venous and capillary plasma concentrations with the line of equality. (**B**) Bland-Altman plot comparing venous and capillary plasma concentrations: (1) lumefantrine, (2) desbutyl-lumefantrine, (3) mefloquine, (4) carboxy-mefloquine, (5) piperaquine. Dotted lines indicate the mean difference and 95% limits of agreement. Pearson’s correlation coefficients (*r*) and *P*-values for no difference are shown.

First, the drug concentrations of venous and capillary plasma samples were directly compared (Fig. 2A). As expected, venous and capillary samples were highly correlated (Pearson’s correlation coefficient: 0.90–0.99) for all antimalarials and their main metabolites. But there were clinically important differences between the two measures, with statistical significance (except for lumefantrine): the 95% limits of agreement were −272 to 236 ng/mL for lumefantrine, −13.9 to 7.2 ng/mL for desbutyl-lumefantrine, −110 to 558 ng/mL for mefloquine, −105 to 81 ng/mL for carboxy-mefloquine, and 0.38-fold to 2.38-fold for piperaquine (Fig. 2B).

### Prediction of venous plasma drug concentration using capillary plasma concentrations

Second, we constructed models for predicting antimalarial concentrations in venous plasma using concentrations in capillary plasma, which combined other demographic and clinical information. The best prediction models for each antimalarial are shown in Table S2. Only sampling time from the first dose improved the prediction of the models.

**Fig 3:**
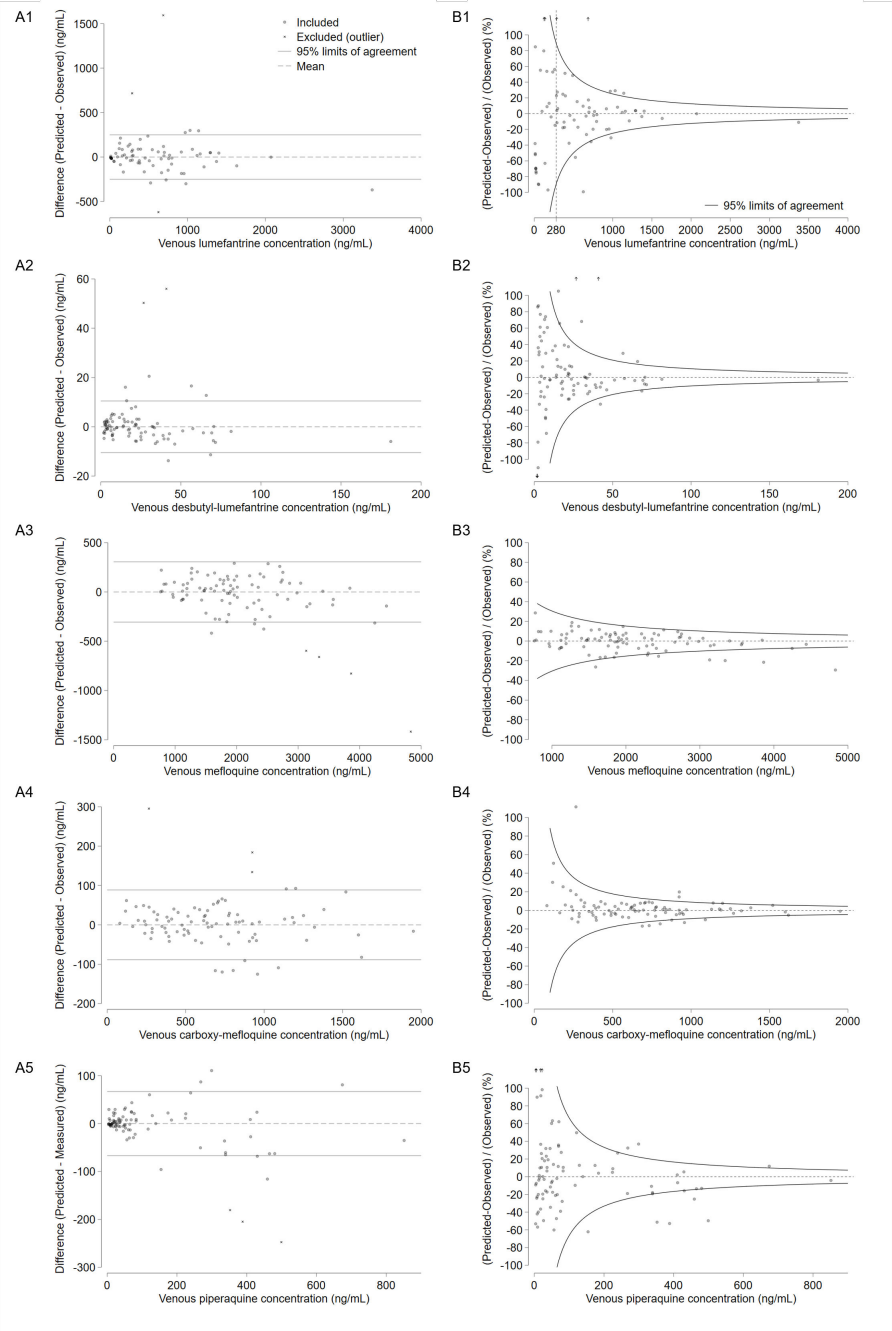
Residual plots of linear regression model predicting the venous plasma antimalarial concentrations by capillary plasma concentrations. The differences (residuals) are shown in the original scale (**A**) or relative to the observed venous plasma concentrations (**B**): (1) lumefantrine, (2) desbutyl-lumefantrine, (3) mefloquine, (4) carboxy-mefloquine, (5) piperaquine. Dotted lines indicate the mean, and solid lines indicate 95% limits of agreement. Arrows indicate observations out of range (>120% or <−120%).

Although adjusted *R*^2^ exceeded 0.95 for all models, the residual plots showed that the 95% limits of agreement were too wide to predict venous plasma concentrations precisely, except for mefloquine and carboxy-mefloquine (Fig. 3). The mean absolute differences between observed and predicted values relative to observed value [and standard deviation (SD) of the residual] were 37.9% (SD 127.5 ng/mL) for lumefantrine, 32.7% (SD 5.4 ng/mL) for desbutyl-lumefantrine, 7.4% (SD 156.0 ng/mL) for mefloquine, 7.7% (SD 45.2 ng/mL) for carboxy-mefloquine, and 36.8% (SD 34.1 ng/mL) for piperaquine. The proportion of venous samples predicted within a ±10% precision range using the concurrent capillary sample was 34% (26/76) for lumefantrine, 36% (32/89) for desbutyl-lumefantrine, 74% (67/90) for mefloquine, 82% (74/90) for carboxy-mefloquine, and 24% (21/89) for piperaquine.

## DISCUSSION

In this study, venous plasma concentrations of mefloquine and carboxy-mefloquine were predicted adequately using concurrent capillary plasma samples. Although drug concentrations in venous and capillary plasma samples were highly correlated, venous plasma concentrations of lumefantrine and piperaquine could not be predicted precisely by the drug concentrations in concurrent capillary plasma samples. Although previous studies used Peason’s correlation coefficient, *R*^2^, or the slope of regression line to assess the correlation between venous and capillary blood concentrations [e.g., lumefantrine (3, 4), mefloquine (5, 6), carboxy-mefloquine (5), and piperaquine (7–9)], correlation is expected. What matters is the agreement between the two measures. However, few studies (4, 7, 9) have commented on whether the agreement was acceptable for clinical or pharmacological use, and only one study, which used capillary whole blood, quantified the agreement (8). Our study is the first to compare antimalarial drug concentrations between capillary plasma and venous plasma taken concurrently and to quantify the agreement.

Lumefantrine concentrations in venous and capillary plasma samples were reported previously to be nearly identical (3). Another study showed that lumefantrine concentration in capillary plasma samples could be 11.9% lower than that in venous plasma samples, based on population pharmacokinetic modeling rather than direct comparisons of the samples taken simultaneously (18). Similarly, 91 pairs of venous and capillary plasma samples from 26 adults were taken to compare the lumefantrine concentrations, showing that the slope of the linear regression (on a natural log scale) was 0.95 (95% CI 0.87 to 1.03) (4). That study concluded, however, that the agreement was “not complete” (4).

The lack of precision in agreement becomes problematic when using capillary plasma samples to predict venous plasma concentrations at the individual level. For example, it is widely accepted that the venous plasma antimalarial concentration on day 7 (e.g., lumefantrine below 280 ng/mL) is associated with a higher risk of recrudescence (treatment failure) (1). We have shown that the day 7 lumefantrine concentrations in venous plasma predicted by capillary plasma concentrations were accurate on average but not precise enough for each pair of samples (95% agreement limits of the error: ±249.95 ng/mL) and only 34% could be predicted with ±10% precision. An alternative threshold (e.g., 355 ng/mL) for capillary plasma (19) can be utilized instead of converting capillary plasma concentrations to venous plasma concentrations.

Two previous studies reported a high correlation of mefloquine concentrations between venous and capillary whole blood samples. The first study using dried blood spot samples showed a high correlation between venous and capillary whole blood concentrations of mefloquine (*n* = 22, *r* = 0.99) and carboxy-mefloquine (*n* = 19, *r* = 0.94) (5). The other study showed that mefloquine concentrations in venous and capillary whole blood were very similar (mean ratio 1.02, 95% CI 0.95–1.09, *n* = 60) (6). Our study confirms that the precision of predicted venous concentrations of mefloquine and carboxy-mefloquine using capillary samples could be acceptable.

The findings of piperaquine concentrations in concurrent venous and capillary samples were inconsistent. One study reported piperaquine concentration on day 7 in capillary plasma was about 1.6 times higher than that in venous plasma (9, 20). Similarly, another study showed that piperaquine in capillary whole blood was 1.66 times (90%, range 0.92–3.03) higher than that in venous whole blood (8). In contrast, another study showed that the median ratio of capillary plasma to venous plasma concentration was 1.08 (inter-quartile range 0.92–1.33) (7), which is close to our finding (median 1.09, inter-quartile range 0.82–1.37). As concluded in the previous studies (7–9), this study suggests that the agreement was not adequate as only 24% of values were predicted within ±10% range of error.

The variability of capillary plasma samples can result from the different extent of mixing of interstitial fluid when capillary blood was squeezed and technical measurement errors (1, 4). Concentration of antimalarial drug in platelets and white blood cells, concentration within malaria parasites, and large differences between red cells and plasma can all contribute to variability in plasma samples. Additionally, capillary blood can be more vulnerable to contamination by antimalarials in the environment while handling samples and treatment drugs (1). The reason why only mefloquine and its metabolite could be predicted precisely was not obvious, but the higher concentrations of mefloquine might be one reason for the small relative difference.

There were some limitations in this study. Firstly, we used plasma samples, so our results may not be comparable to the studies using whole blood samples. The concentrations of piperaquine and mefloquine were reported to be higher in whole blood than in capillary blood (6, 8). For chloroquine, and increasingly for other drugs, whole blood has largely replaced plasma as the matrix of choice. If a satisfactory whole blood assay can be developed, as it can for most of the current antimalarial drugs, then whole blood may be the assay matrix of choice. This also avoids the technically challenging task of separating plasma from the concentrated cells in hematocrit tubes. Secondly, the number of lumefantrine samples with concentrations below the quantification limit was unexpectedly high (14%) despite all the samples being taken on day 7. In pregnancy, the elimination half-life of lumefantrine is shortened (21), resulting in the lower day 7 concentrations. This is one explanation, and one that contributes to the lower lumefantrine efficacy in pregnancy (10, 22, 23). Finally, our plasma samples were stored at −80°C for about 5–10 years. The long-term stability of the frozen plasma samples was not established, although it may not be likely that venous and capillary plasma samples were affected differently during the storage.

### Conclusion

While capillary plasma samples have been promoted as a method to avoid venous plasma samples and the antimalarials concentrations were highly correlated, they were not directly interchangeable. Using prediction models, the precision of agreement was satisfactory only for mefloquine, but not for lumefantrine or piperaquine. Capillary plasma samples can be utilized for pharmacokinetic and clinical studies (24), but the values are not necessarily interchangeable with those derived from venous plasma measurements.

## Data Availability

De-identified participant data are available from the Mahidol Oxford Tropical Medicine Data Access Committee upon request from this link: https://www.tropmedres.ac/units/moru-bangkok/bioethics-engagement/data-sharing.

## SUPPLEMENTAL MATERIAL

Supplemental tables aac.00093-24-s0001.pdf Tables S1 and S2.

ASM does not own the copyrights to Supplemental Material that may be linked to, or accessed through, an article. The authors have granted ASM a non-exclusive, world-wide license to publish the Supplemental Material files. Please contact the corresponding author directly for reuse.
