# Supplementary material for: Comparison of lumefantrine, mefloquine, and piperaquine concentrations between capillary plasma and venous plasma samples in pregnant women with uncomplicated falciparum and vivax malaria
Source: Antimicrob Agents Chemother. 2024 Apr 10;68(5):e00093-24. doi: 10.1128/aac.00093-24 (PMC11064628; doi:10.1128/aac.00093-24)
Supplement: Supplemental tables — Tables S1 and S2. [file aac.00093-24-s0001.pdf]

Table S1. Overall method performance for each antimalarial and their main metabolite

Drug	Lumefantrine			Desbutyl-lumefantrine			Mefloquine			Carboxy-mefloquine			Piperaquine		
QC concentration	33.5 ng/mL	709 ng/mL	15000 ng/mL	2.86 ng/mL	40.6 ng/mL	577 ng/mL	24.2 ng/mL	256 ng/mL	2704 ng/mL	24.2 ng/mL	256 ng/mL	2704 ng/mL	4.50 ng/mL	20.0 ng/mL	400 ng/mL
**Mean value**	33.2	710	14523	2.89	40.6	556	23.4	251	2780	24.7	263	2733	4.43	20.0	387
**SD**	1.51	18.0	322	0.154	1.86	28.8	0.670	4.28	42.8	0.881	7.96	82.2	0.247	0.817	15.5
**RSD (%)**	4.54	2.54	2.22	5.34	4.59	5.17	2.86	1.71	1.54	3.57	3.03	3.01	5.59	4.09	4.01
**LLOQ**	7.77 ng/mL			0.808 ng/mL			7.64 ng/mL			7.64 ng/mL			1.20 ng/mL		

QC: Quality control. RSD: Relative standard deviation. SD: Standard deviation. LLOQ: Lower limit of quantification.

Table S2. The best model predicting venous plasma concentration for each antimalarial drug

Drug<br>Parameter	Coefficient (95% CI)
Lumefantrine	
Capillary plasma concentration	0.96 (0.91–1.01)
Constant	5.47 (-38.14–49.08)
Desbutyl-lumefantrine	
Capillary plasma concentration	0.98 (0.93–1.02)
Constant	-2.68 (-4.35– -1.02)
Mefloquine	
Capillary plasma concentration	1.01 (0.95–1.07)
1/(Sample time in hour)^2	-1.40x10^7 (-2.15x10^7– -6.59x10^6)
LN(Sample time in hour) /(Sample time in hour)^2	3.58x10^6 (1.66x10^6–5.51x10^6)
Constant	48.89 (-60.97–158.75)
Carboxy-mefloquine	
Capillary plasma concentration	0.97 (0.94–1.00)
Sample time in hour	0.13 (0.02–0.24)
Constant	-9.39 (-32.39–13.62)
Piperaquine	
Capillary plasma concentration	0.74 (0.69–0.78)
1/(Sample time in hour)^2	8.55x10^4 (3.41x10^4–1.37x10^5)
Constant	0.38 (-10.39–11.15)

CI: confidence interval, LN: Log natural.
